# How Gaze Direction and Dynamics Affect Visual Resolution

**DOI:** 10.1523/ENEURO.0176-26.2026

**Published:** 2026-09-08

**Authors:** Josselin Gautier, Norick R. Bowers, Martin S. Banks, Austin Roorda

**Affiliations:** ^1^ Herbert Wertheim School of Optometry and Vision Science, University of California, Berkeley, Berkeley, California 94720-2020; ^2^LTSI, Inserm UMR 1099, University of Rennes, Rennes 35042, France; ^3^School of Optometry and Vision Science, University of Waterloo, Waterloo, Ontario N2L 3G1, Canada

**Keywords:** adaptive optics, eye tracking, fixational eye movements, visual acuity

## Abstract

Humans exhibit machine-like eye movements (consistent and repeatable) in space and time while performing demanding acuity tasks. To investigate these, we used an adaptive optics imaging and display system in 6 human subjects (4 females, 2 males) to present ultra-sharp Vernier acuity stimuli briefly every 2 s while simultaneously measuring eye movements, including precisely where on the retina each stimulus fell. We found that drifts and microsaccades combined to confine the landing location of the anticipated stimulus to a tiny retinal region centered on the preferred retinal locus (PRL). The variance of landing location was smallest at the time of stimulus presentation and a few hundred milliseconds after. We correlated where the stimulus fell in space and time with correct or incorrect responses. The PRL and a small area around it, including the anatomical fovea, conferred the best acuity. Acuity declined consistently in the rare events in which the stimulus fell more than 5 minarc from the PRL. We also found that acuity was best when the last microsaccade occurred sufficiently prior to stimulus presentation. Our findings reveal a highly evolved oculomotor system where gaze direction during fixation is rarely far enough from the PRL to compromise visual resolution when a person makes natural fixational eye movements.

## Significance Statement

When humans hold their gaze on an object, their eyes are in constant motion, yet the consequent movement of the image on the retina is not perceived. Using advanced optical methods to measure the exact trajectory of fixated images on the photoreceptor mosaic and the consequent visual performance in a repeated acuity task we reveal a highly evolved oculomotor system. Humans exhibit machine-like control over their ocular fixation, using a combination of drifts and saccades (fast flicks of eye gaze) to maintain the image of fixated objects on a tiny retinal region called the preferred retinal locus (PRL). Only rarely does the gaze ever shift far enough away to compromise visual performance.

## Introduction

**Figure 1.**
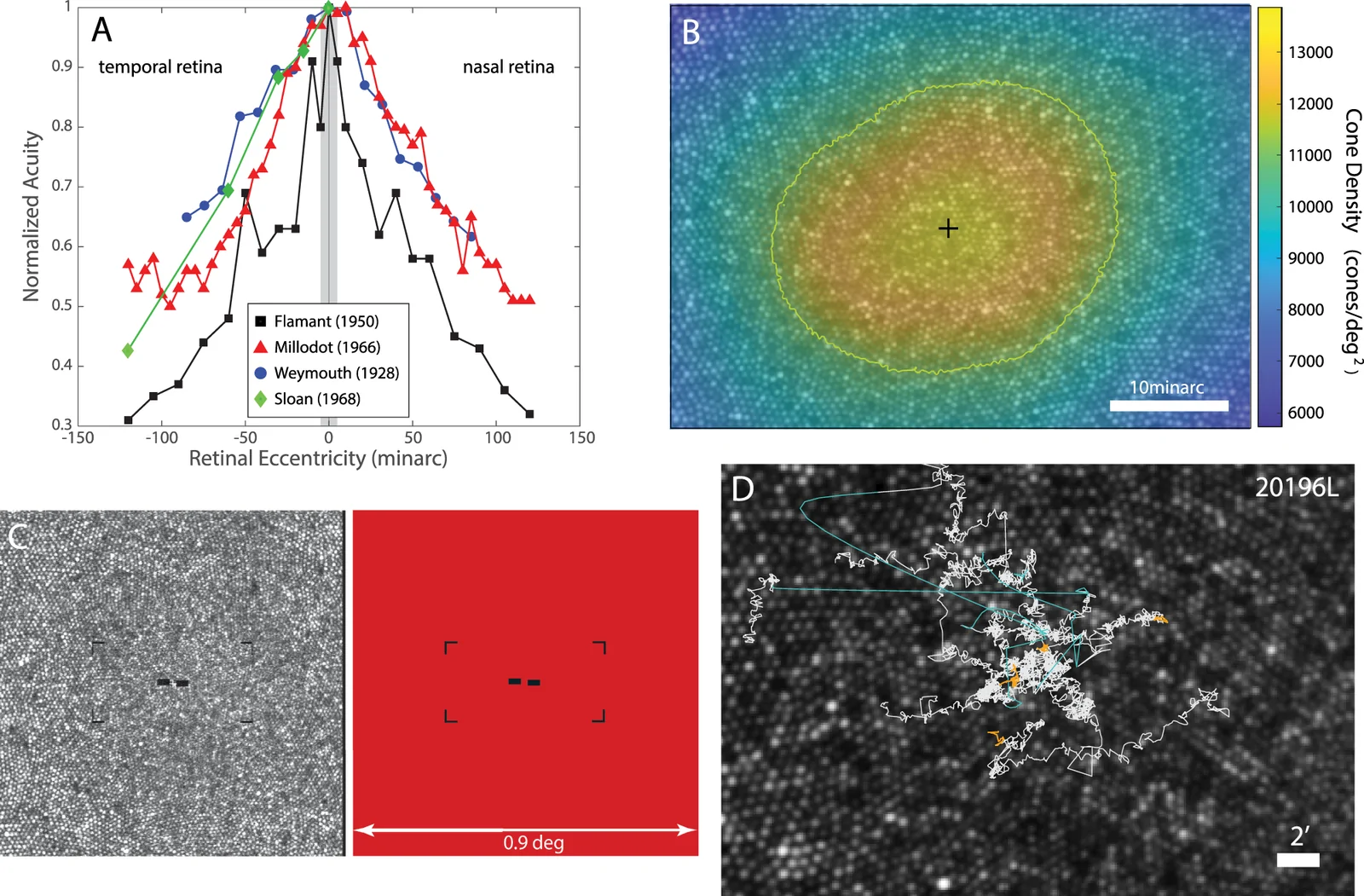
Introduction and methods. ***A***, Measurements of visual acuity as a function of eccentricity are plotted for four previous studies that made measurements near the line of sight. The data have been normalized, so the highest acuities have a value of 1 on the ordinate. Data from the temporal and nasal retina are on the left and right, respectively. Black squares represent data from Flamant (1950): Two-dot separation acuities from one subject. Red triangles represent data from Millodot (1972): Landolt-C acuities averaged from three subjects. Blue circles are data from Weymouth et al. (1928): grating acuities from one subject. Green diamonds are from Sloan (1968): Landolt-C acuities at 1.5 log millilamberts from one subject. The gray rectangle represents eccentricities spanning +/ − 10 minarc from the line of sight, which is the region we focused on in the current study. ***B***, Cone mosaic for one subject. Density (cones per square deg) is represented by color as indicated by the color bar. The green contour represents where density falls to 80% of the peak. The black cross represents the cone density centroid (CDC), which is the density-weighted centroid within the 80% contour. Scale bar indicates 10 minarc. ***C***, The left panel shows one raw adaptive optics, scanning-light ophthalmoscope (AOSLO) frame. The fixation guides and Vernier stimulus are generated by turning off the same 680 nm laser that is used to record the image. Thus the stimulus and fixation guides are encoded directly into the AOSLO frame. The right panel shows how the raster appears to the subject. Field size is $0.9\times 0.9^{\circ }$, fixation guides are 25 × 12.5 minarc apart, and each bar of the stimulus is 0.95 × 1.9 minarc, separated by 0.95 minarc. ***D***, Traces of target position on the retina for five consecutive trials in a representative subject. The stimulus was presented for 34 ms. Its position during that time is indicated by the orange traces. We also plot where it would have fallen on the retina across the whole 2-s trial. White traces represent drifts. Cyan traces represent microsaccades. Scale bar indicates 2 minarc.

Humans, unlike most animals (Walls, 1942; Hughes, 1977; Peichl, 2005), have a specialized retinal region—the fovea—with tightly packed photoreceptors (Osterberg, 1935; Curcio et al., 1987, 1990). Foveal cones each make connections to at least two retinal ganglion cells (Curcio and Allen, 1990; Wässle et al., 1990) and have a magnified representation in visual cortex (Horton and Hoyt, 1991; Wandell and Winawer, 2011). These specializations enable the resolution of finer detail with the fovea compared to other parts of the retina (Weymouth et al., 1928; Ludvigh, 1941; Millodot et al., 1975; Thibos et al., 1987; Banks et al., 1991; Rossi and Roorda, 2010). Figure 1*A* plots data from previous studies showing that acuity (letters, gratings, or Vernier stimuli) falls off precipitously with eccentricity (Weymouth et al., 1928; Millodot, 1972; Westheimer, 1981; Levi et al., 1985). Thus, it is well established that the ability to resolve fine detail is much better at the fovea than elsewhere.

Interestingly, the actual retinal region people use when they fixate a small target—the preferred retinal locus (PRL)—is close to the anatomical foveola, but not exactly on it. High-resolution imaging has revealed that PRLs are, on average, displaced 4 to 10 minarc away from the the cone density maximum (Putnam et al., 2005; Rucci and Poletti, 2015; Wang et al., 2019; Bowers et al., 2021; Reiniger et al., 2021) and are usually displaced upward in retinal coordinates (Putnam et al., 2005; Bowers et al., 2021; Reiniger et al., 2021). The assumption that a fixated stimulus falls on the anatomical fovea and that the eccentricity of a non-fixated stimulus is its distance from the foveal center was reasonable for many studies (Weymouth et al., 1928; Ludvigh, 1941; Millodot, 1972; Levi et al., 1985), is carefully considered in this investigation.

In addition to the PRL’s displacement from the anatomical fovea, the image of the fixated object is also in constant motion due to fixational eye movements (FEM). FEM are characterized by slow drifts interspersed with fast microsaccades (Rucci and Poletti, 2015). It is argued that FEM may impair visual resolution, much like a shaky camera produces a blurred photograph (Arend Jr, 1973; Packer and Williams, 1992). But it is also shown that FEM are beneficial to high-resolution vision because they effectively increasing sampling within a temporal integration window (Averill and Weymouth, 1925; Ratnam et al., 2017; Anderson et al., 2020; Nghiem et al., 2025) and also reshape the spatiotemporal properties by “whitening” spatial energy across the temporal domain (Rucci and Victor, 2015).

Regardless of their role, one consequence of FEM is to cause uncertainty about the actual position of the stimulus on the retina at any moment, possibly leading to a misleading impression that acuity varies less around the center of gaze than it actually does (Intoy and Rucci, 2020). This is potentially solved by stabilizing the acuity target on the retina (Intoy and Rucci, 2020) but, without imaging the retina, one still cannot know where the stabilized stimulus fell again leading to the possibly false impression that acuity plateaus near the fovea.

Researchers have recently used high-resolution retinal imaging combined with psychophysics to better investigate visual performance and retinal eccentricity. Specifically, they investigated visual performance at the PRL or displaced from it. Domdei et al. (2021) used stabilized imagery to position stimuli at the PRL or 6 or 12 minarc from it. They reported that detectability was essentially constant across the region tested: specifically, that detectability was no better at the PRL than at the locus of peak cone density. Ratnam (2017) conducted a letter-acuity task for stabilized stimuli presented at the PRL and up to 10 minarc from it and found no drop-off in performance. Witten et al. (2024) argued that FEM serve to direct the fixated image toward retinal locations with higher cone densities, but they did not test if these higher density regions in the same individuals conferred greater acuity. In summary, using retinal imaging in tandem with either accurate recording or direct control of the stimulated region, researchers have reported that visual sensitivity and acuity plateau across the small retinal region surrounding the PRL and peak cone density.

The motivation for the current work was to employ a more challenging visual task (Vernier acuity) and employ an approach (natural eye-motion) that is better suited to reveal the retinal region that enables the highest resolution.

## Materials & Methods

### Subjects

Six subjects participated, 2 males and 4 females. All self-reported to have normal vision and no ocular disease. Data from one female subject were dropped after learning that they initially misunderstood how to respond in the Vernier task. Of the remaining subjects, average age was 28.2 years. Two (20196 and 20109) were authors. Cycloplegia and mydriasis were induced with drops of 1.0% tropicamide and 2.5% phenylephrine hydrochloride ophthalmic solution. All procedures were approved by the Institutional Review Board at the University of California, Berkeley. Informed consent was obtained from each subject before the experiments.

### Apparatus

The apparatus is an adaptive optics scanning-light ophthalmoscope (AOSLO). It is a custom-built device that can image the retina of one eye while also delivering images to its retina. The system uses adaptive optics to measure and correct for optical imperfections that would otherwise blur the images. Subjects bit into a dental impression plate mounted to an *X*-*Y*-*Z* stage to maintain stable alignment in the system. More details on the system are published elsewhere (Roorda et al., 2002; Mozaffari et al., 2020).

The AOSLO uses different wavelengths for wavefront sensing, imaging, and psychophysics: 940 nm for wavefront sensing and 680 nm for imaging and stimulus-delivery. The scanned field size was $0.9\times 0.9^{\circ }$ and each frame was digitized into 512 × 512 image pixels yielding a pixel size of 6.33 secarc. Video imaging was done at about 30 frames per second.

The 680-nm raster-scanned light formed the display, but because it needed sufficient power for simultaneous imaging, it appeared to the subject as a very bright red square field (estimated to be 5.8 log Trolands Domdei et al., 2017). The brightness level is not expected to compromise visual performance (McMahon and Macleod, 1998). A set of fixation guides at the corners of a 25 × 12.5 minarc rectangle were present in every frame (Fig. 1*C*). Subjects were asked to maintain fixation in the middle of the guides, which should have elicited relatively stable fixation (Steinman, 1965; Thaler et al., 2013). For two frames out of every 60-frame (2 s) sequence, a Vernier stimulus consisting of two horizontal bars appeared at the center of the fixation guides. The bars were 0.95 × 1.9 minarc (9 × 18 pixels) and the horizontal gap between them was 0.95 minarc. The size of the Vernier stimulus was intentionally small so that it could serve as an effective spatial probe to identify the exact retinal region underlying each offset judgment. The fixation guides and Vernier stimulus were presented within this field by turning off the scanning laser at specific time points during the scan (Poonja et al., 2005; Rossi and Roorda, 2010). The same laser that was used for imaging was used to present the decrement stimulus, so the actual stimuli appeared as dark pixels in the recorded image of the retina (Fig. 1*C*). This offers an unambiguous record of the exact location where the stimulus landed, with cellular-level accuracy. The stimulus appeared ultra-sharp to the subject owing to the use of adaptive optics and large pupil (7.2 mm).

### Image- & eye-motion analysis

Incessant eye movements that occur during AOSLO scanning caused unique distortions within each frame of the recorded video. We used versions of previously published algorithms to remove distortions and stabilize each recorded video from each subject (Stevenson and Roorda, 2005). Correcting for these distortions serves two purposes.

First, it enables the summing of multiple frames to provide images with high signal-to-noise. All frames in each distortion-corrected video were added to generate a high-quality image of the foveal cone mosaic. The smallest cones in the center of the fovea could be resolved in all of our subjects. The best-appearing image from one of the videos for each subject was used as their master retinal image. Semi-automated methods were used to label every cone in this image (Li and Roorda, 2007). Cone density maps were computed by integrating all the labeled cones within a 10 minarc circular area around each pixel in the master image (Wang et al., 2019). The CDC was computed as the density-weighted centroid within the contour at 80% of the peak density, as per methods described by Reiniger et al. (2021). An example master retinal image with cone density colormap overlay from one subject is shown in Figure 1*B*.

Second, the byproduct of the distortion correction is a high-speed, high-accuracy eye-motion trace. For the videos, we corrected the distortion at 32 segmented strips across each scanned frame and generated eye-motion estimates at 960 Hz. Each eye-motion trace from each video was annotated for drifts, microsaccades, and blinks using semi-automated, custom software. Segments with bad tracking due to poor image quality were dropped from each trace, but there were few such instances and they were short in duration.

The eye movement traces indicate how a continuously viewed scene would have moved across the retina during fixation. To make each eye-motion trace yield the exact retinal trajectory of the Vernier stimulus, we added a fixed offset to the trace to align its horizontal and vertical location with the horizontal and vertical location of the stimulus on the frames in which it appeared. Figure 1*D* shows a master retinal image with 960-Hz traces overlaid for one video consisting of five consecutive trials. The traces are color coded to show drifts (white), saccades (cyan), and the trace when the stimulus was present (orange). All the data from the complete eye-motion traces and 2,100 trials for each subject were registered to that subject’s master retinal image. All analyses were drawn from that master data set. Despite training, some subjects landed their saccade more than three standard deviations away from the others, probably in an attempt to search for the stimulus when they did not perceive it. Such landing positions were rejected as outliers and not included in the computation of the isodensity (ISO) contours.

**Figure 2.**
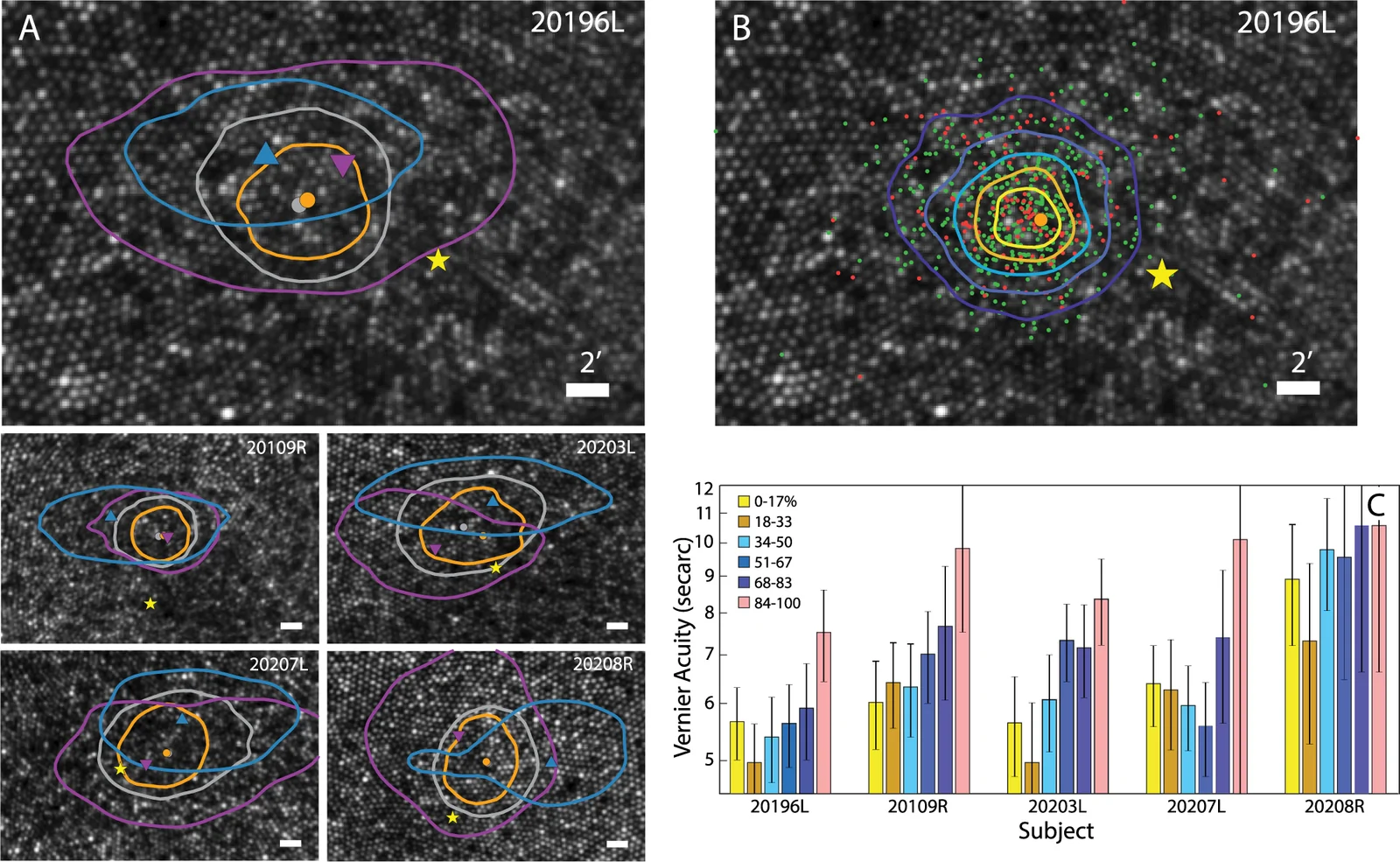
PRLs, ISO contours, and performance. ***A***, PRLs and ISO contours from all five subjects. Gray dots and contours are the PRL and ISOA contours from the entire distribution of target locations for the 2 s trials. Orange dots and contours are the PRL and corresponding ISOA computed from the 34 ms when the target was presented. Purple triangles and contours are computed from the starting position of each microsaccade: the Saccade Starting Position. Blue triangles and contours are computed from the landing position of each microsaccade: the Saccade Landing Position. These two positions are where the stimulus would have fallen if it were presented when the saccade started or ended. Yellow stars are the location of the CDC. Scale bars indicate 2 minarc. ***B***, Stimulus positions and performance. The position of the stimulus across trials is plotted for a representative observer. Green dots represent the retinal loci of the stimulus when the observer responded correctly. Red dots are for incorrect responses. Only the +/ − 6.33 secarc offsets are shown for purposes of clarity. The yellow star indicates the CDC. Yellow, orange, cyan, blue, and purple curves are the ISO contours that contain 17, 33, 50, 67, and 83% of the responses, respectively. Scale bar represents 2 minarc. ***C***, Acuity thresholds based on where the stimulus fell. Different sets of histograms are for different observers. The colors are, respectively, thresholds based on the 17% of positions falling closest to the PRL (yellow), 18–33% (orange), 34–50% (cyan), 51–67% (blue), and 68–83% (purple), and 84–100% (pink). Error bars are standard deviations estimated by bootstrapping.

Figure 2*A* plots the distributions of retinal locations on the master retinal image as ISO contours that encompass 68% of the data points for the entire trajectory (gray), the saccade start points (purple), saccade end points (blue), and the retinal locations where the stimulus landed (orange). We call the stimulus landing locations the functional PRL or fPRL. The peak of the kernel density distribution for each respective scatter plot of data points is indicated by a symbol of the same color. The areas of the isodensity contours (ISOA) encompassing 68% of the scatter plots of the data points are provided in Table 1.

**Table 1.** Subject data

Subj ID	Eye	Vernier acuity (secarc)	ISOA (minarc^2^)				fPRL-CDC (minarc)	Nyquist Limit (c/deg)		Num trials (2,100 max)
			TotPRL	fPRL	SacStart	SacEnd		at fPRL	at CDC		
20196	L	6.29 ± 0.27	55.18	25.68	176.97	69.95	5.87	67.0	68.2	2,087
20109	R	7.46 ± 0.31	34.62	22.99	69.65	101.96	6.23	65.3	68.8	2,007
20203	L	6.98 ± 0.32	102.29	51.70	138.13	132.49	2.94	64.0	64.0	1,989
20207	L	7.40 ± 0.36	98.64	50.67	229.59	141.47	3.87	69.7	70.3	1,975
20208	R	10.76 ± 0.55	77.00	44.04	231.21	120.76	6.59	60.4	62.8	1,954
AVG		7.64	73.54	39.02	169.11	113.33	5.10	65.3	66.8	2,002.4

All averages are arithmetic averages except for Vernier acuity which was converted to logarithms before averaging. TotPRL refers to fixations during whole trial. fPRL refers to fixations when the stimulus was present. SacStart refers to fixations at the beginning of each microsaccade. SacEnd refers to fixations at the end of each microsaccade. Nyquist sampling limit of the cone mosaic in cycles per degree. CDC refers to the cone density centroid. Num Trials indicates the total number of trials analyzed per subject (out of a maximum of 2,100).

### Vernier acuity measurement

We used the method of constant stimuli to estimate the smallest offset yielding reliably correct performance. Upward and downward offsets of 0, 1, 2 and 3 pixels (0, 6.33, 12.66, and 18.99 secarc) between the two bars were presented in pseudo-random order. Data were collected for blocks of 21 trials over the course of a 44-s AOSLO video. One hundred blocks were conducted for each subject for a total of 2,100 trials for each subject. Experimental sessions took about 2 h per subject. The Vernier stimulus was delivered for two frames at the middle of each 2-s trial. In a 2-alternative, forced-choice judgment, the subject indicated on each trial, using a key press, whether the left bar had been higher or lower than the right bar. No feedback as to the correctness of the response was provided. In the rare event that no response occurred within the 2-s window between stimulus presentations, that trial was discarded.

Threshold was estimated by fitting the resulting psychometric data with a logistic function using a maximum-likelihood criterion (Prins and Kingdom, 2018). From the fitted function, we found the positive (left bar up) offset size yielding 75% left responses and the negative (left bar down) offset size yielding 25% left responses. The threshold estimate was the difference in those two sizes divided by two. We also computed standard deviations of the acuities from the psychometric fits using bootstrapping. An example of the psychometric data and the function fit to it is provided in Figure 1-1. The figure shows that the psychometric data provided a reliable estimate of Vernier acuity threshold.

10.1523/ENEURO.0176-26.2026.f1-1 Figure 1-1 **Psychometric data and fitted function.** The proportion of trials in which this subject responded that the left line was higher than the right line is plotted as a function of the offset between the left and right lines. Circles represent the proportion of responses. Error bars are 95% confidence intervals. Blue curve is the fit to the data. Red lines indicate the offset values corresponding to left responses on 0.25 and 0.75 of the trials. It is important to note that proportion correct is not at ceiling when the offset was equal to the pixel size of 6.33secarc. Thus pixel size did not constrain the Vernier-acuity threshold. Download Figure 1-1, TIF file.

## Results

### Spatial analysis

We define the location of the anatomical foveola as the location of the CDC, which corresponds to the centroid of the region of peak density (Reiniger et al., 2021; Fig. 1*B*). The CDC is also labeled on each master retinal image. The fPRL is displaced from the CDC by 5.10 minarc on average (Table 1) and is above the CDC in all cases. These two observations are consistent with previous reports (Wang et al., 2019; Reiniger et al., 2021). The ISOA contour containing 68% of the stimulus landing points does not include the CDC in any of our subjects.

We know from previous studies that FEM differ when observers perform different tasks. For example, drifts become slower and more curved when performing an acuity task compared to simply maintaining fixation on a small target (Intoy and Rucci, 2020). Less drift during acuity tasks generally leads to better performance (Clark et al., 2022), and microsaccades are less frequent in an acuity task (Bowers et al., 2021). From such data, it has been concluded that FEM are fine-tuned during resolution tasks so that the resulting positioning of the upcoming stimulus on the PRL and the reduced motion of the retinal image help maximize acuity (Intoy and Rucci, 2020; Bowers et al., 2021; Clark et al., 2022). We tested this claim by determining how the eyes move before, during, and after stimulus presentation and by measuring the correlation between the movements and performance in the demanding acuity task.

Figure 2*A* shows how our subjects’ eye movements led to less variance of gaze direction when the stimulus appeared compared to before and after its appearance. The contours represent 68% of gaze directions at different times during a trial. Gray contours are the ISOAs from the entire distribution of stimulus locations during the 2-s trials. Orange contours are the ISOAs when the stimulus was present. Purple and blue contours represent the ISOAs respectively at the start and end of microsaccades. A key observation is that the ISOA for the fPRL is the smallest of the four: specifically, the area for the fPRL is 39.02 minarc^2^ on average, which is about half the value of the overall ISOA. This suggests that the entire trajectory of the eye-motion during the task serves to confine the landing position of the anticipated stimulus to a small retinal area. We note that the overall ISOA (gray) is the more conventional definition for the PRL (Castet and Crossland, 2012). The ISOAs for the saccade starting and ending points are larger, their contour plots are elongated horizontally, and the endpoints are displaced vertically from the startpoints (in four of the five subjects). These tendencies are consistent with earlier reports of larger and more frequent horizontal microsaccades (Cherici et al., 2012; Thaler et al., 2013) along with a tendency for microsaccades to have an upward vertical component (Bowers et al., 2021).

We measured Vernier acuity instead of increment sensitivity (Domdei et al., 2021) or letter acuity (Ratnam, 2017) because Vernier acuity has a steeper drop-off with retinal eccentricity than the other tasks (Levi et al., 1985). Thus our task is more likely to reveal a small peak where performance is best if there is one. The Vernier acuity thresholds were derived from subjects’ responses in a forced-choice procedure (Fig. 1-1). The acuities were 7.64 secarc on average, which is consistent with high-quality measures of Vernier acuity (Westheimer, 1981; Levi et al., 1985; Whitaker et al., 1992). The fact that the acuities were very high confirms the effectiveness of the stimulus-delivery system, the fixation guides, and the geometry of the stimuli. The individual and average acuity values are provided in Table 1.

We next computed Vernier acuity as a function of where the stimulus fell on the retina trial by trial. Figure 2*B*, *C* shows the results. Figure 2*B* displays, for a representative subject, the retinal location of the stimulus on trials in which the subject’s response was correct (green dots) and trials in which the response was incorrect (red dots). For illustration purposes, only the trials with the +/ − 6.33 secarc offsets are shown. It also shows contours that contain the 17% of positions that are closest to the fPRL (yellow), 18–33% of the closest (orange), 34–50% (cyan), 51–67% (blue), and 68–83% (purple). There were equal numbers of observations per subject in each region, including 84–100% (no contour). Figure 2-1 shows the data for the other four subjects. Figure 2*C* plots for each subject the acuity thresholds based on the data in each of the six regions. Lower thresholds (i.e., higher acuities) were obtained when the stimulus was closer to the fPRL. The increase in threshold (decrease in acuity) with increasing distance from the fPRL was highly reliable statistically [1-way repeated-measures ANOVA; *F*(5, 50) = 15.48, *p* = 3 × 10^−6^]. A Friedman test (considering the relatively small sample size) also confirmed the effect [*χ*^2^(5) = 17.70, *p* = 0.003], with a large effect size (Kendall’s *W* = 0.71). Finally, paired-comparison tests (post hoc *t*-tests, Holm-corrected) revealed that the effect is due to significant fall-offs in performance for points from 68 to 83% (*p* = 0.042) and 84% or farther from the fPRL (*p* = 0.018). The average acuity decrease from the region immediately around the fPRL (yellow contour) to the 68–83% region was 19% and to the 83–100% region was 44%. Said another way, acuity did not decline significantly until the stimulus fell approximately 5 minarc or farther from the fPRL. All other pairwise comparisons did not reach statistical significance. Thus, during this cadenced acuity task, FEM placed the stimulus in a non-optimal position only about 34% of the time, leading to acuity losses from 19% to 44%.

10.1523/ENEURO.0176-26.2026.f2-1 Figure 2-1 **Stimulus positions and performance for the subjects not shown in Fig. 2B.** The position of the stimulus is plotted for each subject. Green and red dots represent those positions on trials in which the response was correct and incorrect, respectively. Only the +/-6.33secarc offsets are shown for clarity. Orange circles represent the PRL. Yellow stars represent the cone-density centroids (CDCs). The colored contours indicate, respectively, 17% of positions falling closed to the PRL (yellow), 18–33% (orange), 34–50% (cyan), 51–67% (blue), and 68–83% (purple). Download Figure 2-1, TIF file.

**Figure 3.**
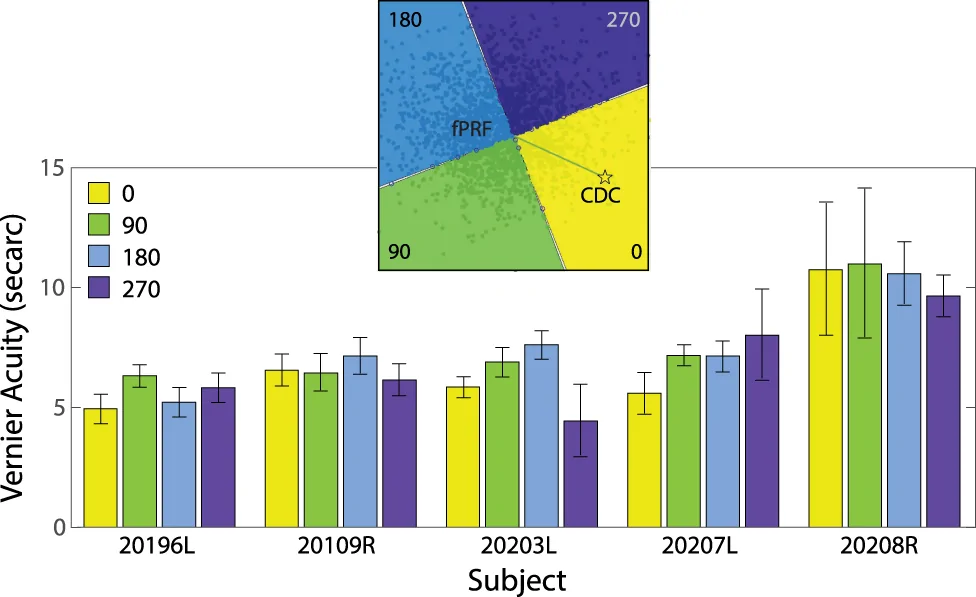
Directional analysis. Visual acuity as a function of direction from the fPRL. The inset shows the four sectors for one subject each subtending 90°. The center line of the yellow sector was directed from the fPRL to the CDC. The center lines of the other sectors were 90, 180, and 270° from the first one (green, blue, and purple, respectively). The dots indicate stimulus positions on all trials. Similar sectors were defined for the other four subjects with the yellow sector always directed from the fPRL to the CDC. The plot shows Vernier acuity thresholds for each of the sectors for each subject. Threshold was estimated from the correct and incorrect responses that occurred for stimuli falling in each sector. Yellow indicates thresholds for the sector toward the CDC. Green, blue, and purple indicate thresholds for the sectors 90, 180, and 270° clockwise from yellow. Error bars are standard deviations estimated by bootstrapping.

Although the ISO analysis (Fig. 2*C*) might suggest that the performance at the CDC is lower (because it is over 5 minarc away from the fPRL, on average), we decided to compare acuity between the fRPL and CDC performance more directly. We did this in two ways. First, we computed percent correct response when the stimulus fell within 1.5 minarc of the fPRL relative to stimuli falling within that distance from the CDC. Proportion correct within 1.5 minarc of the fPRL was 0.790 (STD = 0.18). Proportion correct within the same distance from the CDC was 0.822 (STD = 0.053). Subject 20196 had no trials within 1.5 minarc of the CDC and so was not included in this comparison. There were, of course, many more observations near the fPRL because that location is by definition where stimuli were most likely to fall. Accounting for this, we fit a logistic regression with subject fixed effects and frequency weights (trials per condition). The location effect (fPRL vs. CDC) was not significant, $\hat{\beta }=-0.017$ (log-odds), *z* = −0.08, *p* = 0.940, corresponding to an odds ratio of 0.98 with 95% CI [0.63,  1.53]. Thus, we did not find evidence that acuity differed between the two locations. Second, we performed a sector analysis in which we compared Vernier acuities in four primary directions from the fPRL. The orientations of the sectors were chosen so that one was centered on the CDC, as indicated by the yellow sector in Figure 3. The resulting acuities are shown in the lower part of the figure. Although there were differences between sectors, there was no systematic trend of improved or worse Vernier acuity in the direction of the CDC.

Finally, we asked whether acuity was better for points within 1.5 minarc of the saccade landing position and found that it was not. Thus, Vernier acuity is essentially constant within a small region in the center of the retina even when subjects make natural FEM to position and move the stimulus on the retina.

### Temporal analysis

**Figure 4.**
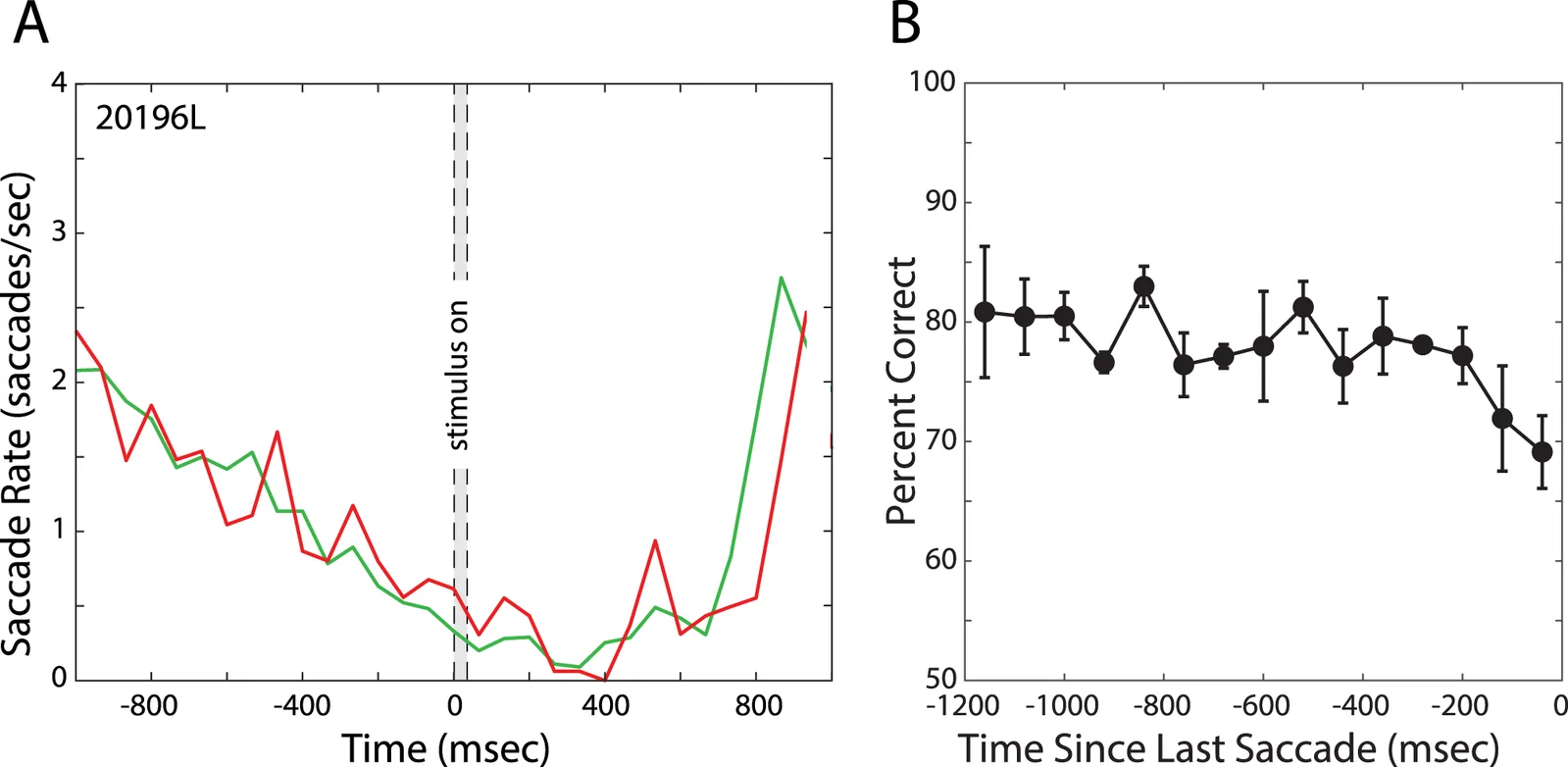
Saccade rate and performance. ***A***, Saccade rate for a representative subject over time. The average number of saccades per second is plotted as a function of time. Stimulus onset is at time 0. The gray bar represents the time the stimulus was presented. The green and red lines represent saccade rate during trials in which the observer responded correctly and incorrectly. ***B***, Last saccade and performance. Percent correct performance in the acuity task is plotted as a function of how much time the last microsaccade preceded stimulus presentation. The data have been averaged across the 5 subjects. Each data point is the percent correct for 80 ms bins centered on the time values on the abscissa. Percent correct was calculated excluding trials with Vernier offsets of +/ − 18 secarc because performance was at ceiling for those trials. Error bars are standard errors of the mean.

Subjects suppressed microsaccades prior to, during, and following the stimulus. Figure 4*A* plots, for a representative subject, the frequency of microsaccades over the 2-s duration of each trial cycle. The time of stimulus presentation is represented by the gray bar and the saccade rate during correct and incorrect trials by the green and red lines, respectively. The microsaccade rate was slightly lower during stimulus presentation on correct trials than on incorrect ones. All subjects exhibited similar behavior (Fig. 3-1): They reduced the number of microsaccades as the time of stimulus presentation approached and continued to do so for about 400 ms after extinction of the stimulus. This was followed by a return to a higher saccade rate as they provided their response and awaited the next stimulus presentation. The greatest suppression occurred about 200–400 ms after the stimulus was extinguished. These findings align with previous reports (Bridgeman and Palca, 1980; Betta and Turatto, 2006; Ko et al., 2010). Additionally, we observed a tendency in all subjects toward lower saccade rates during stimulus presentation on correct trials compared to incorrect ones.

We also examined when the last microsaccade occurred before the stimulus was presented and how that timing affected performance. The last microsaccade usually occurred 300–600 ms before the stimulus appeared. Figure 4*B* plots percent correct as a function of the time of the last microsaccade. It shows how that timing affected performance in the acuity task. There was a fairly consistent change in percent correct as a function of lag with shorter lags being associated with lower percent correct. For lags of 520 ms and less, percentage correct declined (negative slope of −20.8 percent/s [Pearson *r* = −0.861 (*p* = 0.0128)]). The individual subject slopes were all negative and confirmed a significant trend (two-tailed *t*-test, *p* = 0.0056). Thus, making a saccade within 500 ms of stimulus onset hinders performance in a resolution task.

### Spatiotemporal analysis

**Figure 5.**
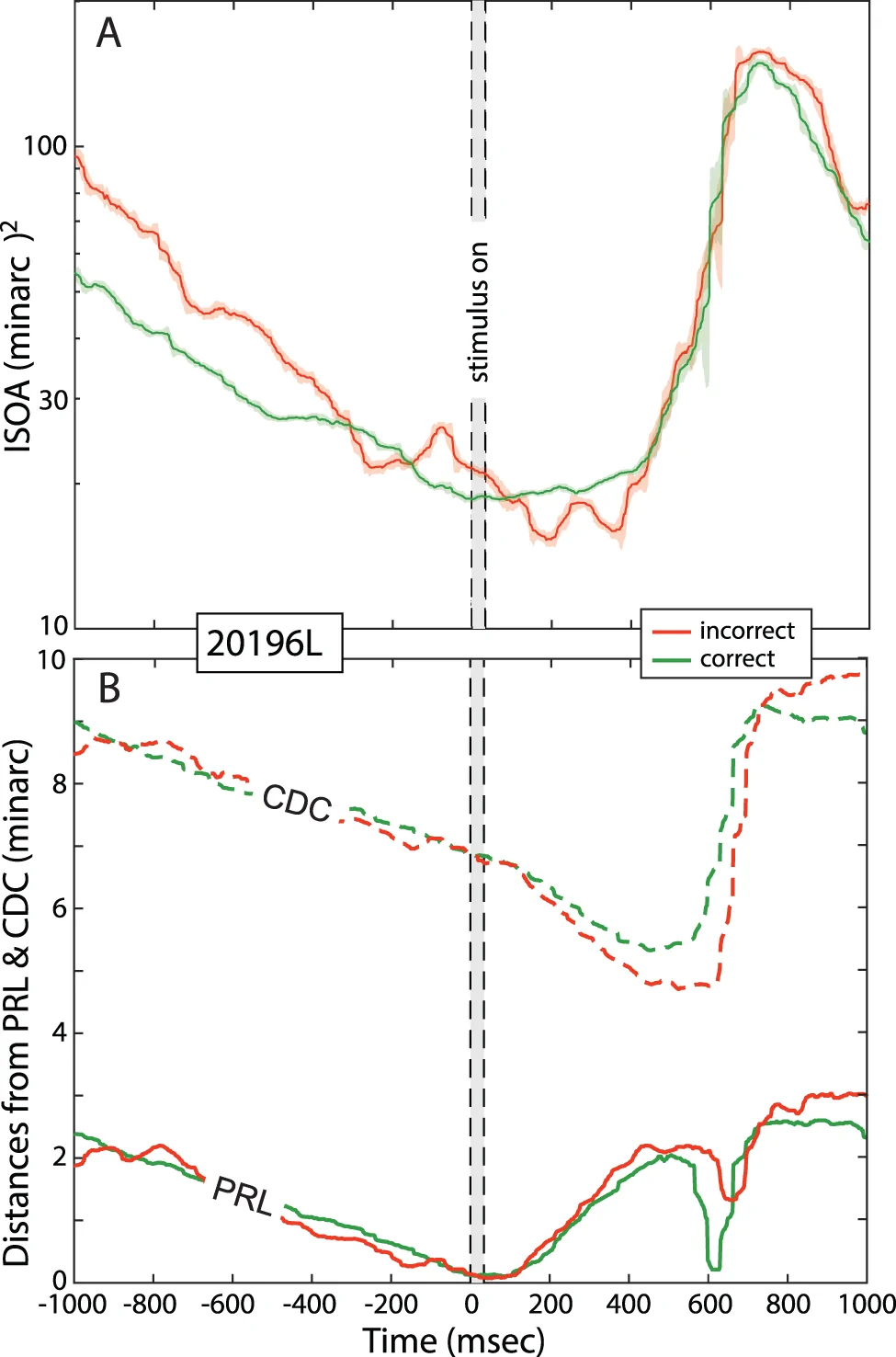
Variation of fixation during the course of a trial. ***A***, ISOA over time. Area of the ISO contour containing 68% of retinal positions is plotted over the 2-s duration of a trial for a representative subject. The time at which the stimulus was presented is represented by the gray bar. The data have been averaged across all 2,100 trials. A running median was applied at each time point over 20 preceding and 20 succeeding time samples. Green and red lines represent contour areas on correct and incorrect trials, respectively. Shaded regions around each line indicate standard deviations. ***B***, Fixation relative to PRL and CDC during the course of a trial. The lower solid curves represent median distance of fixation from the PRL over the duration of a trial for the same subject. The distance goes to zero at the time of the stimulus, by definition. The gray bar represents stimulus presentation time. The data have been averaged across 2,100 trials. The green and red lines represent distance from PRL on correct and incorrect trials, respectively. The upper dashed curves represent the median distance of fixation from the CDC for the same subject. Again green and red represent correct and incorrect responses.

The consistency of fixation strategy during each cadenced trial is illustrated by Figure 5. Figure 5*A* plots the ISOA over the 2-s duration of all trials for a representative subject. The data have been averaged across all trials. The green and red contours represent the ISOAs for trials in which the subject responded correctly and incorrectly, respectively. The ISOA becomes systematically smaller during the 1 s before stimulus onset. We observed that the ISOA was slightly smaller for correct than for incorrect trials. The area remained small for up to 400 ms after the stimulus was extinguished, then became much larger as the subject provided their response and prepared for the next trial. Every subject exhibited this general pattern (Fig. 4-1). The change in ISOA is consistent with the suppression of microsaccades (Fig. 4*A*).

10.1523/ENEURO.0176-26.2026.f4-1 Figure 4-1 **Saccade rate over time.** The average number of saccades per second is plotted as a function of time for the four subjects not shown in Fig. 4A. Stimulus onset is at time 0. The green and red lines represent saccade rate during trials in which the observer responded correctly and incorrectly. Download Figure 4-1, TIF file.

Videos of the ISO-density contours (lines) and their medians (dots) for a representative and the remaining four subjects over the course of the 2-s trials (shown at half-speed) are shown in Movies 1 and 2, respectively. In each movie, the yellow star is the CDC and the time of stimulus presentation is indicated by a change in the color of the ISOA contour. The videos show that ISOAs became smaller and smaller as stimulus time approached and remained small for a few hundred milliseconds after the stimulus was extinguished. Notice that the ISOA never includes the CDC. The graph at the bottom of the figure plots the ISOA containing 68% of the fixations over time. All trials, whether the subject’s response was correct or incorrect, were used to compute the area. The video illustrates compellingly that drifts and microsaccades worked together to confine the stimulus, when it appeared and a bit afterward, to a small region on the retina.

**Movie 1.** Video of the ISO-density contours containing 68% of the data points and their medians (dots) for a representative subject over the course of the 2-sec trials (shown at half-speed). The yellow star indicates the CDC and the time of stimulus presentation is indicated by a change in the color of the ISOA contour. ISOAs become smaller as stimulus time approaches and remains small for a few hundred milliseconds afterward. The overlaid graph plots the ISOA containing 68% of the fixations over time. All trials, whether the subject's response was correct or incorrect, were used to compute the area. [View online]

**Movie 2.** Videos of the ISO-density contours containing 68% of the data points and their medians (dots) for all subjects not shown in Movie 1. The yellow star indicates the CDC and the time of stimulus presentation is indicated by a change in the color of the ISOA contour. For all subjects, ISOAs become smaller as stimulus time approaches and remains small for a few hundred milliseconds afterward. The overlaid graph plots the ISOA containing 68% of the fixations over time. All trials, whether the subject's response was correct or incorrect, were used to compute the area. [View online]

Another indication of the consistency of fixation is illustrated by Figure 5*B*, which plots distances from the fPRL and CDC over the course of trials for the same subject. The solid lines represent distance from the fPRL. As stimulus presentation approached, fixations became systematically closer to the fPRL and remained close as it was presented and then for a few hundred milliseconds after the stimulus was extinguished. The dashed lines represent distance from the CDC. Again as the stimulus time approached, fixation became closer to the CDC, but never as close as it was to the fPRL. As shown in Figure 2*A*, the fPRL was between the saccade landing point and CDC, so movement toward the fPRL also decreased distance to the CDC. The other subjects exhibited similar behavior (Fig. 5-1).

10.1523/ENEURO.0176-26.2026.f5-1 Figure 5-1 ISO **area over time for the subjects not shown in Fig. 5A.** The area of the ISO contour containing 68% of retinal positions is plotted over the 2sec duration of a trial. The time at which the stimulus was presented is represented by the gray bar. The data have been averaged across the 2100 trials the subjects encountered. For each time a running median was computed over the 20 preceding and 20 succeeding time samples. The green and red lines represent contour areas on correct and incorrect trials, respectively. The shaded regions around each line indicate standard deviations. Download Figure 5-1, TIF file.

10.1523/ENEURO.0176-26.2026.f5-2 Figure 5-2 **Fixation relative to PRL and CDC during the course of a trial for the subjects not shown in Fig. 5B.** The solid lower contours represent the median distance of fixation from the PRL the duration of a trial. The time at which the stimulus was presented is represented by the gray bar. The data have been averaged across the 2100 trials the observers encountered. The green and red lines represent distance from PRL on correct and incorrect trials, respectively. The dashed upper contours represent the median distance of fixation from the CDC. Again green and red represent distance on correct and incorrect trials, respectively. Download Figure 5-2, TIF file.

We computed the median trajectories of the eye over the course of the 2-s trials. Movie 3 is a 3D animation of the trajectory for a representative subject. The remaining subjects are shown in Movie 4. The 3D plot has time flowing from the top to the bottom. The green and red traces are the trajectories on trials where the subject’s response was correct and incorrect, respectively. The orange column shows the position of the fPRL. The plot illustrates the consistency of FEM when performing a visual resolution task. At the beginning of a trial, this subject fixates above and to the left of where the stimulus will appear. As stimulus presentation approaches, consistent drift down and to the right occurs; the drift brings the fPRL toward where the stimulus will appear. The drift continues in the same direction as the stimulus is presented and for a couple hundred milliseconds after the stimulus has been extinguished. The substantial decrease in trajectory variability (represented by the transparent green and red disks) during the drift shows that the eye’s trajectory during the drift is much more consistent than its trajectory near the start and end of the trial. The variability of the drift appears to be slightly less for correct than for incorrect ones. This observation is supported by the fact that ISOA is smaller at the time of stimulus presentation for correct than incorrect trials (Figs. 5, S4). Movies 3 and 4 show that every subject exhibited this general pattern: consistent downward drift before, during, and after stimulus presentation. The horizontal component of the drift, however, varied from subject to subject. The trajectories near the end of a trial and the beginning of the next one were dominated by microsaccades and were less consistent than the drifts.

**Movie 3.** Animated plot of the median eye trajectories over the course of the 2-sec trials for a representative subject. The 3D coordinates are horizontal position and vertical position in minarc and time in msec. A 3D coordinate was calculated for every time sample in each trial. The median of those points was computed for each time sample and contours were drawn from those points using a Savitzky-Golay filter to smooth the data. The green and red traces are the trajectories on correct and incorrect trials, respectively. The trajectories start at the top, 1,000msec before stimulus appearance, and end at the bottom, 1,000msec after. The transparent green and red ellipses represent standard errors every 76msec. Trials in which the Vernier offset was +/ − 18 secarc have been excluded because performance was at ceiling on those trials. Including them does not, however, affect these trajectories noticeably. The orange cylinder represents the fPRL. [View online]

**Movie 4.** Animated plots of the median eye trajectories over the course of the 2-sec trials for all subjects not shown in Movie 3. The 3D coordinates are horizontal position and vertical position in minarc and time in msec. A 3D coordinate was calculated for every time sample in each trial. The median of those points was computed for each time sample and contours were drawn from those points using a Savitzky-Golay filter to smooth the data. The green and red traces are the trajectories on correct and incorrect trials, respectively. The trajectories start at the top, 1,000msec before stimulus appearance, and end at the bottom, 1,000msec after. The transparent green and red ellipses represent standard errors every 76msec. Trials in which the Vernier offset was +/ − 18 secarc have been excluded because performance was at ceiling on those trials. Including them does not, however, affect these trajectories noticeably. The orange cylinder represents the fPRL. [View online]

## Discussion

### Fixation strategy & acuity

Humans adopt a machine-like (i.e., consistent and repeatable) fixation strategy that places an anticipated stimulus on a very specific retinal location: the fPRL. This locus is significantly smaller than the overall PRL (Table 1, Fig. 2*A*). Recent studies using retinal imaging and stabilized stimuli reported that visual sensitivity and acuity are not detectably better at the PRL than at the anatomical fovea or CDC (Ratnam, 2017; Domdei et al., 2021). We re-examined this by using a unique methodology to look for changes in performance near the foveal center. We chose a hyperacuity task—Vernier acuity—which is more affected by retinal eccentricity than other tasks such as letter acuity (Levi et al., 1985). And we allowed subjects to make natural fixational movements during a cadenced task, which enabled them to position the stimulus and its motion on the retina in a way that presumably optimized performance.

We observed a measureable drop in acuity as the stimulus landed farther from the fPRL, but the drop was small and not statistically significant until the stimulus was more than 5 minarc from that location (Fig. 2*C*). We did not detect a significant difference in acuity between the fPRL and the anatomical fovea. More trials might reveal a difference, but it is expected to be very small.

The direction in which one fixates is voluntary, but the FEM that occur while fixating are not (Poletti et al., 2017). This is evidenced by the fact that humans can maintain steady gaze, but are generally unaware of the drifts and microsaccades during that gaze (Poletti et al., 2010). FEM are only visible under special conditions such as those that give rise to the jitter after effect (Murakami and Cavanagh, 1998) or the Ouchi-Spillmann illusion (Spillmann, 2013). Thus, fixation is not guided from conscious awareness of how and when it deviates. Yet our data show that fixation is sufficiently good to maintain high and consistent performance in a demanding task. Fixational movements led to a significant drop in performance on 34% of trials with the largest drop on only 17% of them. This suggests that one’s control of fixation is more precise than their ability to perceive an error in fixation. This point is supported by a study that found that awareness of gaze direction was 40% less precise than the actual spread of fixation (Kilpeläinen et al., 2021). It is sensible for the oculomotor system to enable a narrower spread of fixation than conscious behavior reveals because if fixation were guided by conscious awareness of gaze direction, performance in the acuity task would have been worse than we observed. And observers would consciously experience fixation errors that the oculomotor system did not correct.

The observation of more precise oculomotor behavior than perceptual behavior is analogous to the findings that people make accommodative responses to imperceptible changes in retinal image blur (Kotulak and Schor, 1986) and make vergence eye movements to imperceptible binocular disparities (Duwaer and Van Den Brink, 1981). Thus, oculomotor behavior that is more precise than perceptual awareness may be a general principle.

### Role of microsaccades

Microsaccades are thought to redirect gaze toward the intended fixation target (Poletti et al., 2013), but we find on a fine scale that they do not. Two observations support this claim.

1. The ISOA for microsaccade landing points is about three times larger than the ISOA for the fPRL (Fig. 2*A*). This means that the drift following a microsaccade serves to direct gaze toward the fPRL. This is illustrated by the trajectories in Movies 3 and 4 that show drifts in a fairly constant (but different for different subjects) direction as the stimulus approaches, is presented, and then extinguished.
2. Most subjects exhibit micronystagmus during fixation (Bowers et al., 2021). Inter-saccadic drifts usually cause a downward change in gaze direction. The oculomotor system takes this into account by making upward microsaccades that direct the anticipated stimulus slightly above the fPRL rather than toward it. This behavior is evident in Movies 1–4.

If the end point of microsaccades placed the stimulus in a more optimal location for vision, we would have observed better acuity near the saccade landing points; we did not.

### PRL versus anatomical fovea

The fPRL was above (5 of 5 subjects) and nasal (4 of 5 subjects) relative to the anatomical foveola, or CDC, in retinal coordinates (Fig. 2), consistent with previous reports (Wang et al., 2019; Bowers et al., 2021; Reiniger et al., 2021).

It has been reported that drift movements tend to redirect images toward the CDC (Witten et al., 2024). If the region of greatest cone density actually conferred a functional advantage, the fPRL should be located there. But it is not. In fact, in all subjects the stimulus rarely landed at the CDC. Figures 5*B* and S5 show that drifts do tend toward the CDC, but they never bring the stimulus closer to the CDC than the fPRL before, during, or after stimulus presentation. The observation that drifts tend to move toward the CDC is instead a consequence of the fact that the fPRL lies between the saccadic landing point and CDC (Fig. 2*A*). Thus a movement toward the fPRL is also a movement toward the CDC.

Reiniger et al. (2021) suggested that the upward displacement of the PRL (downward in world coordinates) relative to the CDC is advantageous in the natural environment. They pointed out that scene elements above the current fixation point are generally farther than the fixated element, and that elements below fixation are generally nearer (Sprague et al., 2015; Gibaldi and Banks, 2019). The vertical displacement of the PRL from the CDC means that scene elements above the direction of gaze are imaged onto the CDC. Reiniger claimed that more distant elements create more power at high spatial frequencies than near elements and that having the region of greatest cone density directed toward those more distant parts enables more accurate measurement of image features there. Unfortunately, there are two problems with this hypothesis.

First, farther parts of natural scenes do not have greater power in high spatial frequencies than nearer parts. The power spectra of natural images tend to fall with the square of spatial frequency: $P(f)=\frac{1}{f^{2}}$ (Field, 1987; Tolhurst et al., 1992; Simoncelli and Olshausen, 2001). Images with such power spectra are scale-invariant; i.e., their statistical properties do not change if one changes the scale at which the images are observed (Field, 1987). Thus, this part of Reiniger’s hypothesis is not true for images created by the natural environment.

Second, we observed that acuity—a measure of sensitivity at high spatial frequencies—is actually no better at the CDC than at the fPRL or in the direction of the CDC compared to other directions from the fPRL. So the greater receptor density at the peak does not confer a resolution advantage compared to the fPRL. (Note that the increased density at the CDC compared to the fPRL would only confer a 2% improvement at best; Table 1.) Therefore, this part of the hypothesis is not supported by our data.

Thus questions remain.

First, why is the fPRL generally displaced from the CDC? When we began this project we thought that we might find that acuity is better at the fPRL than in the region of highest cone density. If this were true, it would help us understand why fixations are directed toward the small and consistent fPRL. But we did not observe such an increase at the fPRL, so our data do not tell us why the fPRL is generally displaced from the region of highest cone density.

Second, why is the fPRL usually displaced upward from the foveal center in retinal coordinates? We have no persuasive answer, but it is interesting to note that the PRL in patients with central scotomas is usually also displaced upward from the foveal center (Crossland et al., 2005; Greenstein et al., 2008; Kolawole et al., 2025). Perhaps whatever drives the visual system to have an upward displacement in people with compromised vision is the same as what drives a small upward displacement in those with normal vision.

### Are human FEM optimal?

There is no simple answer to this question.

We find that microsaccades and drift work in concert to place an anticipated stimulus within a small retinal region, and that performance at or near this fPRL is better than in other nearby regions (Fig. 2*C*). Considering that vision is improved by motion (Rucci et al., 2007; Ratnam et al., 2017) and hampered by temporal proximity to microsaccades (Fig. 4*B*), the combination of drifts and microsaccades reported here may offer a functional advantage. Microsaccades direct the eye’s gaze to a location that anticipates the subsequent drift epoch that both positions the expected stimulus at the fPRL and maintains a finite drift speed. The spatial extent of FEM is so small that the cost of fixation instability has little consequence for visual performance. Specifically, in our demanding visual task, fixation instability caused only a 44% drop in performance (averaged across subjects) on only 17% of trials (Fig. 2*C*). This exquisite control of gaze is executed without conscious awareness.

We also find that the timing of microsaccades matters. When they occur too close in time to stimulus onset, performance suffers (Fig. 4*B*).

But some of our observations seem inconsistent with the idea that FEM optimize performance. For example, the greatest suppression of microsaccades occurs after the stimulus has come and gone (Figs. 4*A*, S3), which seems non-optimal. But during natural viewing, humans make two saccades or microsaccades per second (Yarbus, 1967; Steinman et al., 1973), which means that stimuli remain in a fairly constant retinal position for a bit less than 500 ms. Perhaps our observation of maximum suppression 200–400 ms after stimulus presentation reflects a strategy to suppress eye movements for the duration of a typical natural fixation. It is also interesting to note that the time required to maximize visual acuity is about 400 ms (Baron and Westheimer, 1973).

The fact that the fPRL is displaced from the region of peak cone density (CDC) also seems non-optimal. But the drop in linear cone density from the CDC to the fPRL is only 2% (Table 1), so the expected loss in resolution due to fixating slightly off the CDC is trivial. Moreover, the mapping between the central retina and higher visual areas, such as visual cortex, is strongly affected by the post-natal migration of cones toward the retinal center and the migration of retinal neurons away from that region (Hendrickson and Yuodelis, 1984; Yuodelis and Hendrickson, 1986). It is thus perhaps not surprising that the retinal region for preferred fixation in adults is not identical to the region of highest cone density. Indeed, it is remarkable that they are so close to one another.

## Synthesis

Reviewing Editor: Niko Busch, Westfalische Wilhelms-Universitat Munster Decisions are customarily a result of the Reviewing Editor and the peer reviewers coming together and discussing their recommendations until a consensus is reached. When revisions are invited, a fact-based synthesis statement explaining their decision and outlining what is needed to prepare a revision will be listed below. The following reviewer(s) agreed to reveal their identity: Lappe Markus. Note: If this manuscript was transferred from JNeurosci and a decision was made to accept the manuscript without peer review, a synthesis may not be available.

# Review Synthesis

This manuscript was transferred to eNeuro from the Journal of Neuroscience, where it was reviewed by three expert reviewers. The authors substantially revised the manuscript in response to these reviews before submitting to eNeuro. I invited an independent expert to review this new version, and we both concur that the revisions are substantial and have improved the quality of the manuscript. Reviewer #1 has only a few suggestions for rewording and typos, which should be easy for the authors to address.

# Reviewer 1

This manuscript reports date from a vernier acuity task presented at highest resolution near the CDC of the foveola. The experiment uses cutting-edge technology and appears to be competently performed. The results are mostly descriptive. They provide interesting findings regarding the CDC and the preferred locus of fixation. My comments are only about the presentation and discussion:

Some observations on the paragraph starting on line 36: First, the second sentence states that "people consistently place the target near the anatomical foveola, but about 4-10 minarc away". Should it be ".. NOT near the foveola, BUT ..."? Second, arcmin is more common than minarc as abbreviation for minutes or arc, at least in my reading. Should that be changed? Lastly, the statement in the last sentence "that the eccentricity of a non-fixated stimulus is its distance from the fovea is presumably false" is maybe a bit harsh if the difference is only 4-10arcmin.

Line 56: "whether" should be "where".

Line 81: brightness level

Lines 158 to 172: this seems to be more appropriate for the introduction than for the results section.

Line 261: please specify tendency. Was this something you tested and it was just not significant, p=0.51 or so? Or is this simply from inspecting Figure 4A?

Lines 264ff: this paragraph feels inconsistent. You write that the last microsaccade usually occurred 300-600msec before the stimulus but then, in Fig. 4B you show performance when the last microsaccade occurred 100 or 200 ms before the stimulus. Then you report a marginally significant trend yet conclude with a strong statement about hindering performance. This should be rephrased. The issue is mentioned again in line 416, which should also be rephrased.

Line 332: "Fixation can be voluntary, but its properties in a given task are not". What does that mean? Are the properties of a fixation involuntary? What are the properties? How does one know whether they are involuntary or not? I find the entire following paragraph quite speculative and unclear.
